# Effects of Red Rice or Buckwheat Addition on Nutritional, Technological, and Sensory Quality of Potato-Based Pasta

**DOI:** 10.3390/foods10010091

**Published:** 2021-01-05

**Authors:** Carola Cappa, Monica Laureati, Maria Cristina Casiraghi, Daniela Erba, Maurizio Vezzani, Mara Lucisano, Cristina Alamprese

**Affiliations:** 1 Dipartimento di Scienze per Gli Alimenti, la Nutrizione e l’Ambiente, Università Degli Studi di Milano, Via G. Celoria, 2-20133 Milano, Italy; monica.laureati@unimi.it (M.L.); maria.casiraghi@unimi.it (M.C.C.); daniela.erba@unimi.it (D.E.); mara.lucisano@unimi.it (M.L.); cristina.alamprese@unimi.it (C.A.); 2 Zini Prodotti Alimentari S.p.A, Via Libertà 36, 20090 Cesano Boscone, Milano, Italy; mv@pastazini.it

**Keywords:** dumpling, gnocchi, gluten free pasta, fiber content, starch digestibility, cooking behavior, color, texture, liking predictors, consumer acceptability

## Abstract

This work investigates the effects of red rice (R) or buckwheat (B) flour addition on nutritional, technological, and sensory quality of potato-based pasta (gnocchi). Three gluten-free (GF) and three conventional (C) samples were produced in an industrial line without any addition or with 20% R or B. R and B addition significantly (*p* < 0.05) reduced starch content and increased fat amount and ready digestible starch fraction (potential higher glycemic impact). R addition significantly (*p* < 0.05) worsened GF pasta structure, increasing solid loss in cooking water (5.4 ± 1.2 vs. 4.1 ± 0.5 g/100 g pasta) and reducing product firmness (408 ± 13 vs. 108 ± 2 N). B addition resulted in intermediate consistency (243 ± 8 N), despite the highest total fiber content and weight increase during cooking. Similar trends were found in C samples, indicating a better texturizing capacity of B in comparison to R. Samples without any addition were the most liked (C = 67.4 and GF = 60.6). Texture was the major contributor to liking: uniform structure and firm texture were positive predictors of liking, whereas a granular and coarse matrix contributed negatively. The outcomes of this research can be useful in developing GF potato-based pasta for consumers focused on healthier foods and for industries willing to better valorize their products.

## 1. Introduction

The increase in celiac disease and other allergic reactions to gluten has opened new market opportunities for pasta producers, especially in the sector of fresh products, in which the gluten-free offering is still limited. The removal of gluten represents a challenge for good quality products, because it is responsible for the well appreciated pasta structure. In gluten-free pasta, structure is assured mainly by starch, whose gelatinization degree plays an important manufacturing role [1,2]. In fact, the use of pregelatinized starch ingredients allows the application of a standard pasta production process, whereas non-pregelatinized starch sources require gelatinization to occur during processing. Usually, proteins, hydrocolloids, and emulsifiers are also included in the formulation to improve gluten-free dough workability and quality of the final product [3]. Nutritional properties of gluten-free pasta are not comparable to those of conventional products, because of the reduced levels of dietary fiber, resistant starch, and protein, with higher glycemic index and starch digestion rate [4].

Among fresh pasta products, potato dumplings are very popular in many countries and they are prepared in different way [5]. The Italian version is called “gnocchi”; it mainly consists of potato (fresh or dehydrated), to which wheat flour and salt are added; eventually, eggs, emulsifiers, and preservatives can be used in the recipe [6]. In order to improve nutritional properties of conventional and gluten-free gnocchi, different strategies have been proposed, such as the addition of quinoa and amaranth flours [7], green coffee extract [8], navy bean flour, and meat [9]. In this context, the enrichment with red rice or buckwheat flours could also have a positive effect.

Red rice is a pigmented variety of rice (*Oryza sativa* L.) with beneficial health effects due to the antioxidant activity of bioactive compounds such as phenolic compounds, anthocyanins, and proanthocyanidins, which are associated with protection against chronic diseases [10]. A number of papers demonstrated the anti-oxidant, anti-diabetic, anti-hyperlipidemic, and anti-cancer activity of pigmented rice varieties, which are thus gaining popularity among consumers. However, texture and palatability of pigmented rice are poor and, thus, the consumers’ acceptance is low [11], justifying the limited number of studies on red rice enriched pasta [12,13].

Buckwheat (*Fagopyrum* spp.) is a pseudocereal belonging to the family of the *Polygonaceae*, with a more balanced amino acid composition and, thus, biological value higher than that of most cereals. It can safely be consumed by people suffering from celiac disease and it is rich in constituents important for human health, such as dietary fiber, antioxidants, minerals, and vitamins [14]. Moreover, buckwheat has a high level of resistant starch (27–33.5%), which can help in modulating blood glucose and lipid levels, regulating intestinal microbiota, and reducing obesity [15]. The use of buckwheat in bread, cookies, and pasta formulations, both conventional and gluten-free, has been extensively studied [14,15], however the effects of its addition in gnocchi recipes have not yet been evaluated.

The aim of this study was to investigate the effects of red rice or buckwheat flour addition (20%) on the nutritional, technological, and sensory qualities of conventional and gluten-free quick-frozen gnocchi, produced by a turbo-cooking technology. This thermal technology, patented by VOMM Impianti e Processi S.p.A. (Rozzano, Italy), causes the starch gelatinization during gnocchi production, allowing to obtain a good final product without including pregelatinized ingredients in the formulation [6].

## 2. Materials and Methods

**Figure 1:**
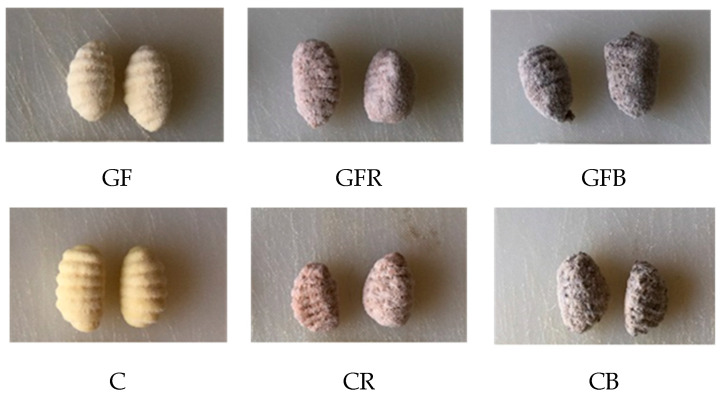
Gluten free (GF) and conventional (C) potato-based pasta samples without or with 20% addition of red rice (R) or buckwheat (B) flour.

Three gluten-free (GF) and three conventional (C) samples of potato-based pasta (i.e., gnocchi) were produced without or with 20% addition of wholemeal red rice (R; Distretto rurale “Riso e Rane”, Cassinetta di Lugagnano, Italy) or wholemeal buckwheat (B; Molino Filippini S.r.l., Teglio, Italy) flour (Figure 1). All of the other ingredients were provided by Zini Prodotti Alimentari S.p.A. (Cesano Boscone, Italy).

The potato-based pasta recipe was defined according to literature data [6] and Zini Prodotti Alimentari S.p.A. (Cesano Boscone, Italy) experience. GF reference sample was made of water, rice flour, dehydrated potato (24 g/100 g), corn flour, and salt; C reference sample contained water, semolina flour, dehydrated potato (33 g/100 g), and salt. All samples were produced by Zini Prodotti Alimentari S.p.A. (Cesano Boscone, Italy) in an industrial line by a turbo-cooking technology (VOMM^®^ Impianti e Processi S.p.A., Rozzano, Italy) followed by individual quick-freezing at −35 °C [6]. Samples were stored at −18 °C till their characterization.

ABTS (2,2-azino-bis(3-ethylbenzothiazoline-6-sulphonic acid) diammonium salt, A1888), Trolox (6-hydroxy-2,5,7,8-tetramethylchroman-2-carboxylic acid, 238,813), potassium persulphate (dipotassium peroxydisulphate, P-5592), pepsin (P7000; ≥250 U/mg), pancreatin (P7545; 8xUSP), invertase (I4504; ≥300 U/mg) and amyloglucosidase (A7095; ≥260 U/mL), were purchased from Sigma Chemical Co. (St. Louis, MO, USA) and chemicals at analytical grade from Merck KGaA (Darmstadt, Germany).

### 2.1. Pasta Cooking Conditions

For nutritional and technological evaluation, pasta samples were cooked in boiling unsalted tap water (1:10 pasta:water ratio) for their optimal cooking time (OCT) and drained for 1 min. For sensory evaluation, samples were cooked at OCT in salted tap water (salt: 10 g/L) in order to make the evaluation more similar to common consumption. The OCT of each sample was defined according to preliminary sensory tests: GF and C, 120 s; CR, 100 s; GFR, GFB, and CB, 90 s.

### 2.2. Nutritional Quality Evaluation

Raw gnocchi samples were ground in a mixer (Bimby VM 2200, Thermomix, Worwerk, Wuppertal, Germany) for 5 min at maximum speed and characterized in terms of composition. Moisture and ash content were evaluated by the official gravimetric methods, lipids were extracted with a mixture of ethyl ether and petroleum ether (2:1) using a Soxhlet apparatus and nitrogen content was detected according to the Kjeldahl method [16]. Proteins were calculated using 6.25 as the nitrogen/protein conversion factor. HPLC with an ion exchange column combined with a pulsed amperometry detection system was used to evaluate soluble sugars [17]. Soluble and insoluble fibers were evaluated by an enzymatic-gravimetric procedure [16,18]. The antioxidant capacity was measured by the ABTS^∙^ assay and expressed as Trolox equivalents (Trolox standard concentrations were 2–22 µmol/L; calibration curve, *r* = 0.994) [19]. All of the evaluations were run in triplicate.

Cooked gnocchi were ground by using a screw-type kitchen grinder, for the analysis of in vitro starch digestibility [20,21]. The method measures the rate of digestion through a series of proteolytic and amylolytic enzymatic attacks under controlled conditions of temperature, pH, viscosity, and stirring speed, simulating the different digestive steps that take place in vivo. Based on the HPLC analysis of the glucose released after 20 min (G20) and 120 min (G120), the fractions of ready (RDS) and slowly (SDS) digestible starch were calculated. The sum of RDS and SDS is indicative of the starch digestible in the small intestine and it is defined as available starch (AvSt). The ratios of RDS and SDS over AvST were also calculated. The digestibility tests were conducted in duplicate and repeated 4 times (*n* = 8).

### 2.3. Technological Quality Evaluation

Color of R and B was evaluated by using a Minolta Chroma Meter II (Minolta, Osaka, Japan) with standard illuminant C, on flours (approximately 30 g) levelled in petri dishes. Results were expressed in the CIE L*a*b* space as L* (lightness; from black (0) to white (100)), a* (from green (−) to red (+)), and b* (from blue (−) to yellow (+)) values. The particle size distribution of the flour samples (50 g) was evaluated by means of the analytical sieve shaker Octagon Digital (Endecotts Ltd., London, UK), by using 4 certified sieves (openings: 90, 125, 250, and 500 µm). Five fractions were collected after sieving for 10 min at amplitude 6 in the presence of 3 plastic spheres (diameter: 3.0 cm) on each sieve, to make the sifting of the fine particles easier. Triplicate measurements were performed for each sample and results were expressed as g/100 g for each fraction.

Raw gnocchi were characterized in terms of weight, surface color (using a colorimeter Minolta Chroma Meter II; Minolta, Osaka, Japan), and geometrical indices (by image analysis according to literature data [2]). Sample images were acquired at 300 dpi resolution using a flatbed scanner (HP SCANJET8300; Hewlett-Packard Development Company, Palo Alto, CA, USA) and covering gnocchi with a black box to amplify the contrast between the objects and the background and to prevent light losses. Images were processed using a dedicated software (Image Pro-Plus v. 4.5.1.29, Media Cybernetics Inc., Rockville, MD, USA) in order to measure sample area, width, and length. For each samples, fifteen randomly-selected raw gnocchi were analyzed.

Cooked gnocchi were evaluated after being cooked at their OCT and cooled in an airtight container for 25 min at room temperature in order to ensure a complete cooling as temperature can affect the texture properties. Cooked samples were characterized in terms of surface color and geometrical indices (as previously reported for raw sample), weight increase, by weighing gnocchi before and after cooking, and solid loss into the cooking water, by evaluating the dry matter of the cooking water (dried at 105 °C, to constant weight). All the measurements were done in triplicate, cooking fifteen gnocchi in each replicate. Gnocchi texture was assessed with a TA-HDplus Texture Analyzer (Stable Micro Systems, Surrey, UK) equipped with a 10-blade Kramer shear cell and a 250-N load cell. The Texture Exponent TEE32 V 3.0.4.0 software (Stable Micro System, Surrey, UK) was used to control the instrument and for data acquisition. Cooked gnocchi (98 ± 10 g) were compressed, sheared, and extruded through the bottom openings of the Kramer cell by the blades moving at 2 mm/s speed, simulating chewing. The maximum force (N) reached during the shear/extrusion test was extrapolated from the stress–deformation curve as an index of the product hardness. Seven replicates were carried out for each sample.

### 2.4. Sensory Quality Evaluation

Ninety-six consumers (36 males and 60 females, age range: 19–64 years, mean age 30.3 ± 11.8) took part in the experiment. All subjects reported to like gnocchi and to consume them at least once in a month. Participants with allergies or intolerances towards ingredients present in the formulations were excluded from the evaluation. All subjects gave their written informed consent prior to the beginning of the study and they were instructed to refrain from smoking, eating, and drinking (except water), in the hour before tasting. The study protocol was approved by the Ethical Committee of the University of Milan. The study was conducted in agreement with the Italian ethical requirements on research activities and personal data protection (D.L. 30.6.03 n. 196) and according to the principles of the Declaration of Helsinki.

The tasting sessions were organized over two consecutive days in the sensory laboratory of the Department of Food, Environmental, and Nutritional Sciences (University of Milan, Milan, Italy) designed according to the International Organization for Standardization (ISO) guidelines [22]. Eight rounds were organized in the 11:00 a.m.–2:00 p.m. time slot, each comprising 12 subjects. For each round, the samples (300 g) were cooked in 3 L of salted water (salt: 10 g/L) and tasted with tomato sauce (40 g/100 g; provided by Zini Prodotti Alimentari S.p.A., Cesano Boscone, Italy) in order to make the evaluation more similar to common consumption. Samples were prepared immediately before each tasting session and served monadically (approximately 20 g by sample) in white plastic dishes coded with 3-digit numbers. The presentation order was balanced according to Latin square to limit carry-over effects [23].

Two methods were applied: a hedonic test to have insights on the overall liking of each sample and the Check-All-That-Apply (CATA) method, which is a simple approach to gather information about consumers’ perception of the sensory characteristics of food products [24]. With this method, consumers are asked to taste the products and to answer a CATA question by selecting from a list of descriptors all the terms that they consider appropriate to describe each of the samples. In the present study, the descriptors were chosen in a preliminary test involving 6 untrained subjects who tasted the six samples and generated a list of 12 terms: 6 for texture in mouth (firm, coarse, rubbery, soft to be chewed, grainy/pieces, adhesive/sticks to teeth) and 6 hedonic terms related to appearance (pleasant and unpleasant appearance), taste (pleasant and unpleasant taste) and texture (pleasant and unpleasant texture). The terms were selected in order to be easily understood by consumers. The number of terms chosen is in line with the number suggested by the literature, i.e., between 10 and 40 terms [25].

Prior to the beginning of the session, participants were instructed about the overall methodology and received a brief explanation of the CATA terms. Then, they were invited to taste the samples in individual sensory booths under normal light conditions. Each consumer was informed about what he/she was going to taste (e.g., “You are going to taste a sample of gnocchi added with buckwheat”). For each sample, at first participants rated their overall liking using an unstructured linear scale anchored at the extremes with “Extremely disliked” (left of the scale, corresponding to 0) and “Extremely liked” (right of the scale, corresponding to 100), then, they were asked to select all the descriptors suitable for describing that sample. The position of CATA attributes in the list was randomized across participants but fixed for each participant [26]. Participants were instructed to drink a sip of water between samples tasting.

### 2.5. Data Analysis

Nutritional and technological results were expressed as mean ± standard deviation (SD) values. All data were subjected to one-way analysis of variance (ANOVA), followed by the Least Significant Difference (LSD) test to identify significant differences between the samples (*p* ≤ 0.05). The statistical analysis was carried out using STATGRAPHIC_Plus for Windows v. 5.1 (StatPoint Inc., The Palins, VA, USA).

Sensory data were processed by a mixed ANOVA model performed on liking data considering subjects as a random factor and samples as fixed factor. The LSD test was used to compare the samples. For the CATA questions, the frequency of mention for each term was determined by counting the number of subjects that used that term to describe each sample. Cochran’s Q test was performed for each of the 12 terms to evaluate significant differences among samples.

To study the relationship between CATA questions, technological properties of cooked gnocchi and liking data, Partial Least Square Regression (PLSR) analysis was performed [27,28]. PLSR models both the X- and Y-matrices simultaneously to find the variables in X that best predict the variables in Y. The PLSR components are referred to as factors or latent variables or latent structures. In PLSR models, scores and loadings express how the samples and variables are projected along the model factors [29]. CATA questions and technological variables were considered as the X matrix and average liking scores of each product as the Y matrix. Data were standardized (i.e., scaled to unit variance) prior to modeling and full cross validation was chosen as the validation method. A correlation loadings plot was used to find variables with less than 50% explained variance which were left out of the model [30]. This only resulted in the omission of one technological variable, i.e., solid loss.

SAS software v. 9.4 (SAS Institute Inc., 2012, Cary, NC, USA) and The Unscrambler X v. 10.3 (CAMO, Oslo, Norway) were used as statistical software packages. A *p*-value ≤ 0.05 was chosen as the threshold for statistical significance.

## 3. Results and Discussion

### 3.1. Nutritional Quality

Proximate composition of gnocchi is reported in Table 1. The amount of water added to the formulation is a critical parameter affecting the quality of the final product and approximately 53 g/100 g of water (corresponding to 49–54 g/100 g of product moisture) was indicated as a good amount for a formulation based on corn flour, rice flour, and dried potatoes [6]. Accordingly, the moisture content of GF and C was 54.7 and 53.3 g/100 g, respectively, whereas gnocchi containing R or B generally showed a higher moisture content (up to 58 g/100 g) as they required more water during pasta production to ensure a good dough workability through industrial machines, this may be due to the presence of wholemeal flours having a higher fiber content. Similar moisture values (58 and 61 g/100 g) were found in the literature for gnocchi containing amaranth and quinoa flours, respectively [7].

As expected, for both conventional and GF products the addition of wholemeal R and B flours tended to increase the levels of some nutrients (i.e., lipids, protein, and dietary fiber) while decreasing the starch content. In particular, the addition of B and R significantly increased fat content to about twice the reference samples, due to the lipids presents in the two flours used. In conventional gnocchi (sample C), the addition of both flours to the formulation resulted in a significant reduction (about 50%) of the soluble sugar content. Only the enrichment with B flour led to a significant increase in the level of total fiber in both conventional and gluten free products, mainly represented by the insoluble fraction. It is worth mentioning that the total fiber contents achieved in GFB and CB (>3%) allows one to report on the label the nutritional claim—“Source of fiber”—in accordance with the European Regulation 1924/2006 about nutrition and health claims provided on food products. The addition of whole buckwheat, a pseudocereal known to possess a high antioxidant potential [19], increased the antioxidant capacity of GFB and CB, probably promoting their protection from oxidation during storage.

**Figure 2:**
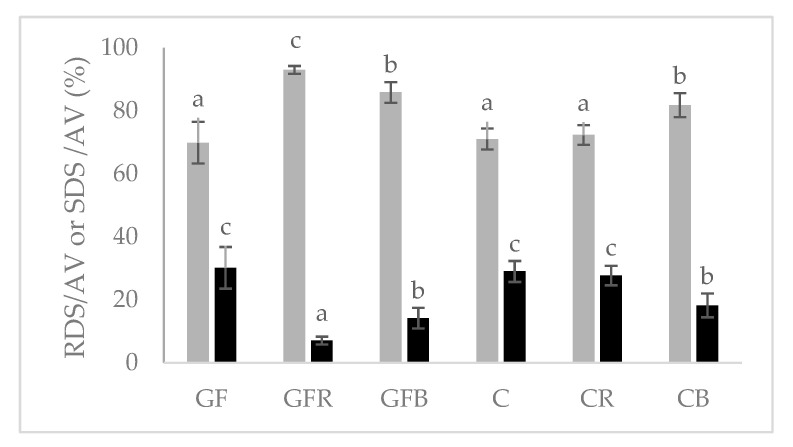
Rapidly (grey bar) and slowly (black bar) digestible starch fractions in cooked gnocchi. RDS, rapidly digestible starch; SDS slowly digestible starch; AV, total available starch. For each parameter, bars with different letters are significantly different (*p* < 0.05).

Regarding the starch quality data shown in Figure 2 evidence that the addition of B and R flours led to changes in the rate of starch digestion in comparison to reference products (C and GF). In particular, the addition of red rice flour in conventional products did not exert significant effects, while in GF gnocchi it increased by about 20% the fraction of rapidly digestible starch and reduced to about a third the fraction of slowly digestible starch, thus suggesting a potential increase in post-prandial glycemia of the enriched products compared to the GF reference. In fact, the RDS fraction is directly related to the glycemic response of the product itself [20,31], while the insulinemic response appears inversely related to the SDS fraction [32]. In addition, recently, the European Food Safety Authority (EFSA) has approved a health claim regarding the role of SDS in the control of post-prandial blood glucose [33]. The different effect of R addition in the two types of reference products could be attributable, at least in part, to the different impact of rice starch on the structure: in GF the presence of rice starch weakened the structure of the finished product [34], while in traditional gnocchi, where the structure is maintained by the presence of gluten, the “destructuring” effect of rice starch was not detected. Differently, the use of buckwheat flour, affected the rate of starch digestion in both types of gnocchi, promoting a greater presence of starch that is rapidly digestible and a reduced level of the slowly digestible, more evident in GF products (RDS +16%; SDS −15%) compared to conventional ones (RDS +10%; SDS −11%), thus likely increasing the glycemic impact of both products. This effect is probably attributable to the presence of a higher fiber content (mostly insoluble), which could interfere with the formation of a compact gluten matrix [34] thus, promoting a greater accessibility of starch to digestive enzymes. In GF products, the higher RDS fraction (>starch accessibility to digestion) is likely to be due to a “destructuring” effect attributable to the presence of fiber that tied water during kneading, thus, compromising the proper distribution of water and interfering with the gelatinization of the dough starch.

### 3.2. Technological Quality

The technological evaluation of raw and cooked gnocchi is summarized in Table 2 and Table 3, respectively.

The color of food is one of the first aspects noticed by the consumer and it can affect its acceptability [35]. Besides the production process, that in the present study was kept constant for all the newly-made gnocchi, pasta color is mostly due to the ingredient used (e.g., type of flour and degree of milling). Color of unconventional flours (R and B) was evaluated: As expected, R flour was characterized by high redness and yellowness values (a* = 4.7 ± 0.2; b* = 10.3 ± 0.3), whereas B had a white-yellow color (a* value around zero; b* = 5.7 ± 0.3). Lightness values (L*) were 70.6 ± 0.5 and 74.8 ± 1.4 for R and B flour, respectively. Consequently, as reported in Table 2, GFR and CR gnocchi were significantly redder than the other samples that had a* values ranging from −5.5 to 0.9. The presence of buckwheat (i.e., GFB and CB samples) caused a reduction of lightness in comparison to the conventional sample and intermediate a* and b* values. Tiny differences were noticed between gluten free and conventional samples characterized by high values of lightness (L* = 76–77) and yellowness (b* = 23–25) values. Similar L* values (77.5 and 63.39–78.34) were reported for reference gnocchi [7,36]. Yellow color in fresh pasta is generally considered an important quality attribute and both GF and C gnocchi showed values in agreement with literature data (b* = 18.5–24 [35] and b* = 22.3 [7]).

As Italian gnocchi are recognized by the consumers for their unique shape (Figure 1), the geometrical indices of raw samples were detected. Gnocchi showed an area ranging from 447 to 534 mm^2^, a width of 18.7–22.7 mm, and a length of 29.9–32.7 mm. No significant differences were noticed between conventional and gluten free references suggesting that ingredients and process conditions were appropriate also in absence of gluten. The addition of wholemeal flours turned out in significant (*p* < 0.05) bigger samples probably due to the fiber swelling and the higher amount of water added that modify the viscosity of the dough and, therefore, the product shaping.

Color evaluation of cooked samples (Table 3) reflected the differences noticed in raw samples; indeed, a strong correlation (r > 0.98, *p* < 0.0005) among L*, a*, and b* values of raw and cooked samples was found, suggesting that R and B flours can be effectively used to confer particular color even to the cooked product. The addition of R and B flours led to a significantly (*p* < 0.05) higher weight increase both in gluten free and conventional samples, maybe due to the higher presence of fiber (Table 1) that binds more water during cooking. All the sample maintained their shape during cooking and they increase principally in length, as evidence in literature [1]. GFR showed also the highest area increase during cooking (3% vs. 0–2% of the other samples, data not shown). Contrary to expectations, no significant (*p* < 0.05) difference among cooking loss values were found, except for GFR that showed a higher solid loss confirming that the addition of unconventional ingredients, such as red rice flour, to a gluten free matrix is more difficult than for conventional samples. The amount of solid loss in cooking water, in fact, is widely used as an indicator of pasta quality: low amounts of residue indicate high cooking quality [1,6,35]. In general, according to literature data [37], all the enriched gnocchi showed good cooking quality having cooking loss < 6 g/100 g. In order to limit the cooking loss, a valuable strategy could be the addition of milk and eggs to the formulation as suggested in a previous study reporting cooking loss < 1 g/100 g even if quinoa and amaranth flour were added to the recipe [7]. In fact, egg proteins can ensure cohesiveness of the dough, mainly when heated [38]. Furthermore, no-forming gluten proteins can create an alternative structure preventing cooking loss [1], and proteins can interact with other compounds, such as starch or albumins, preventing starch leaching [39,40]. According to literature data [36], gnocchi is preferred to be quite thick after cooking and they should not disintegrate even if slightly overcooked. No target consistencies are known based on literature data and many different tests can be performed to investigate gnocchi texture. In the present study, both instrumental texture (Table 3) and sensory texture acceptance (Table 4) were investigated. The hardness of cooked samples evaluated by Kramer test was significantly (*p* < 0.05) affected by the different flours used: both gluten free and conventional gnocchi made with rice flour were 74 and 40% less firm than the references, whereas gnocchi made with buckwheat flour showed intermediate hardness, but in any case, lower than the references (−40% and −22% for gluten free and conventional products, respectively). Similarly, gnocchi made with quinoa and amaranth were reported to be less hard and springy than the commercial ones [7]. As previously mentioned for cooking loss, textural parameters could also be affected by the presence of fiber (which may lead to the formation of discontinuities inside pasta structure [41]), protein matrix [38], and starch organization [1]. Also flour particle size distribution could affect gnocchi texture, since literature data indicate that pasta made of fine (138–165 µm) rice flour is sticky and less hard than sample made with bigger flour [42], whereas rice flour particles < 63 µm improve noodle texture due to a rapid retrogradation of starch and consequently an increase in gel firmness [43]. R flour used in the present study had the following particle size: 1% ≤ 90, 90 < 17% ≤ 125, 125 < 33% ≤ 250, 250 < 41% ≤ 500, and 8% > 500 µm; B flour was characterized by a higher amount of small (<90 µm) and large (>500 µm) particle size: 29% ≤ 90, 90 < 16% ≤ 125, 125 < 30% ≤ 250, 250 < 11% ≤ 500, and 14% > 500 µm.

### 3.3. Sensory Quality

**Figure 3:**
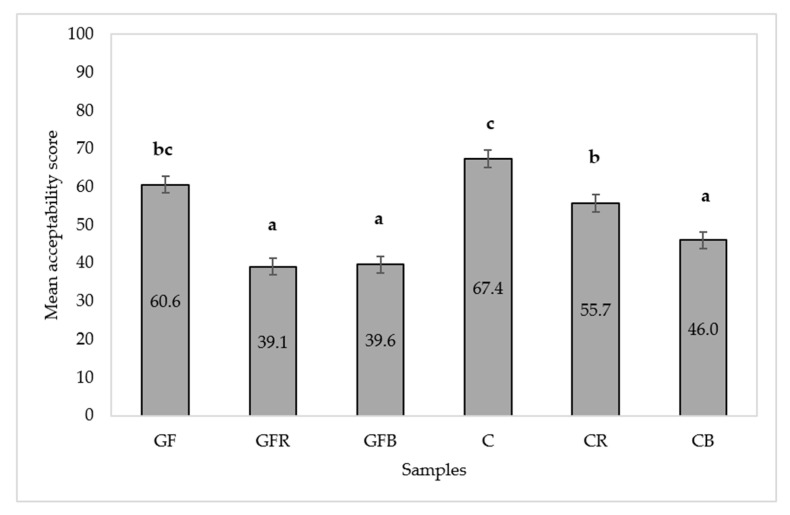
Mean acceptability scores with standard error of the mean. Different letters indicate significant differences according to Least Significant Difference (LSD) post-hoc test.

Understanding the drivers of liking and disliking of GF products is important considering that food appearance, aroma, taste, and texture play a key role in food appreciation and, thus, in its consumption. In fact, dissatisfaction with both the availability and the hedonic dimension of GF products has a decisive impact on the non-compliance with gluten-free diet [44]. There was a significant difference among gnocchi samples in terms of overall acceptability (*p* < 0.0001). The multiple comparison test (Figure 3) revealed that C sample obtained the highest score but it was statistically comparable to GF sample which, in turn, was not significantly different from CR sample. The CB, GFB, and GFR samples were comparable to each other, but scored significantly lower and were significantly less appreciated than the other samples.

The frequency table of terms checked by consumers to describe the gnocchi samples is reported in Table 4. Significant differences (*p* < 0.0001 for all items) were found in the frequency mention for all CATA items. The most appreciated samples (C and GF) were associated more frequently with descriptors such as pleasant appearance, taste, and texture. On the other hand, characteristics such as grainy and coarse texture were used to describe samples with integration of buckwheat, while the integration of rice gave samples a sticky texture, especially to sample GFR (71.9%), which was also perceived as softer (56.3%) than the other samples.

**Figure 4:**
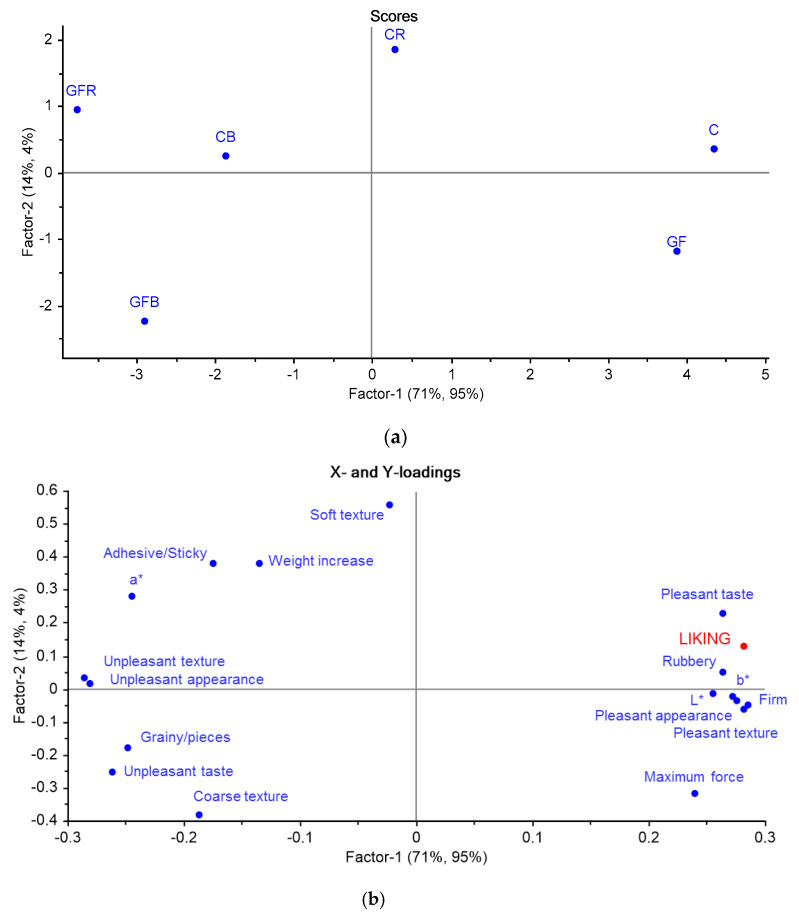
Scores (**a**) and loadings (**b**) plots obtained by the Partial Least Square Regression (PLSR) model of the six gnocchi samples based on CATA questions, technological variables, and liking.

Scores and loadings plots from PLSR performed on technological variables, CATA items and liking are reported in Figure 4a,b. The purpose of this calculation was to establish which technological variables and sensory attributes predict the preference for the samples. The first factor explains respectively the 71% and 95% of the variation in Y, while the second factor accounts for respectively the 14% and 4% of variation. The Y variable (LIKING) is located in the upper right quadrant (Figure 4b). As the first factor explains almost all the information in the model, variables having a positive coordinate on the first factor show a direct correlation with preference, while variables with a negative coordinate on factor 1 are negatively correlated to preference. Texture was a major contributor to liking and rejection of the samples. Firm and rubbery texture properties were positive predictors of liking, whereas a granular and coarse matrix contributed negatively to liking. Firmness perceived by consumers was positively correlated with maximum force. L* and b* colorimetric coordinates also contributed positively to liking, while a* contributed negatively (Figure 4b). Comparing the scores (Figure 4a) and loadings (Figure 4b) plots, gnocchi without addition (C and GF) were the most preferred, because they were characterized by higher firmness and by bright/yellow color (as expressed by L* and b* parameters). Samples with rice addition (GFR and CR) had a soft and adhesive texture, which was related to a higher weight increase after cooking, and a higher intensity of red color as expressed by the a* parameter. These properties were disliked by consumers. GF products are often reported being of poorer sensory quality compared to conventional products, especially with regards to texture due to the lack of viscoelastic properties imparted by gluten. GF pasta is generally characterized by high stickiness, low firmness, and is prone to important cooking loss [1,45]. Hydrocolloids and gums have been reported to improve firmness and mouthfeel sensations of GF pasta formulations because they are able to create a network which contributes to a better perceived texture [46]. Samples with buckwheat addition (GFB and CB) were also rejected by consumers due to their grainy and coarse texture likely due to the higher amounts of particles having dimensions higher than 500 µm and to a higher fiber content, which also imparted an unpleasant taste and appearance to these samples. Previous studies on wheat bran enriched pasta showed that fibers elicit negative sensations in consumers such as dark color, bitter taste, and a coarse texture, which can make food unpalatable [47]. Consumers like to be in full control of the food placed in their mouth. In this context, food containing unexpected lumps or hard particles are usually rejected for fear of gagging or choking [48]. GF products lack of many important nutrients including dietary fibers because they are usually obtained from refined flour and/or starches that are not enriched or fortified [46]. Buckwheat is a valuable source of fiber, therefore, its incorporation in GF formulations is important and should be optimized. The present findings indicate that texture properties of both conventional and GF formulations added with buckwheat should be improved by reducing particles size thus making the matrix more uniform and palatable.

## 4. Conclusions

The addition of 20% of rice and buckwheat whole meal flour both to conventional and gluten free gnocchi caused important changes in the nutritional, technological, and sensory properties of the cooked products. Rice flour addition highly modified GF pasta structure, increasing solid loss in cooking water and reducing product firmness, while the same flour added to conventional gnocchi determined a limited increase of cooking losses and a lower softening after cooking and a higher appreciation, though an excessive stickiness represented the main defect.

Buckwheat addition has allowed for a fiber content in both conventional and GF gnocchi higher than 3% and, thus, the possibility to report on the label the nutritional claim—“Source of fiber”—in accordance with the European Regulation 1924/2006. Furthermore, cooking losses were not affected by buckwheat addition indicating a better texturizing capacity in comparison to rice. On the other hand, great attention has to be paid to the particle size of wholemeal buckwheat as a high number of particles greater than 500 µm (mainly derived from the seed coat grinding) produces negative sensations in the consumers, thus reducing the product acceptability.

## Figures and Tables

**Table 1:** Nutritional composition (g/100 g) and antioxidant capacity (mmol TEAC/kg) of raw gnocchi.

	GF	GFR	GFB	C	CR	CB
Moisture	54.7 ± 0.2 ^bc^	58.1 ± 0.2 ^d^	56.6 ± 0.6 ^d^	53.3 ± 0.1 ^b^	51.1 ± 0.9 ^a^	54.7 ± 0.9 ^c^
Ash	1.31 ± 0.04 ^a^	1.35 ± 0.19 ^a^	1.38 ± 0.14 ^ab^	1.14 ± 0.04 ^a^	1.68 ± 0.05 ^c^	1.67 ± 0.14 ^bc^
Lipids	0.26 ± 0.01 ^a^	0.47 ± 0.06 ^b^	0.55 ± 0.04 ^b^	0.83 ± 0.06 ^c^	1.40 ± 0.10 ^e^	1.15 ± 0.07 ^d^
Proteins	4.2 ± 0.4 ^a^	4.4 ± 0.2 ^a^	4.6 ± 0.3 ^ab^	6.4 ± 0.1 ^cd^	5.5 ± 0.3 ^bc^	6.6 ± 0.8 ^d^
Starch *	40.3 ± 0.1 ^e^	36.6 ± 0.2 ^d^	31.2 ± 0.1 ^b^	33.9 ± 0.4 ^c^	34.5 ± 0.7 ^c^	29.5 ± 0.1 ^a^
Sugars	0.26 ± 0.01 ^a^	0.34 ± 0.02 ^a^	0.36 ± 0.08 ^a^	4.70 ± 0.49 ^c^	2.77 ± 0.24 ^b^	2.66 ± 0.25 ^b^
TDF	0.8 ± 0.3 ^a^	1.3 ± 0.3 ^ab^	6.0 ± 0.5 ^d^	1.7 ± 0.1 ^b^	1.8 ± 0.1 ^b^	4.8 ± 0.1 ^c^
IDF	0.6 ± 0.1 ^a^	1.0 ± 0.3 ^a^	5.4 ± 0.4 ^c^	1.0 ± 0.2 ^a^	1.0 ± 0.2 ^a^	4.2 ± 0.2 ^b^
SDF	0.2 ± 0.1 ^a^	0.3 ± 0.1 ^a^	0.7 ± 0.1 ^b^	0.7 ± 0.1 ^b^	0.8 ± 0.1 ^b^	0.6 ± 0.1 ^b^
TEAC	2.13 ± 0.18 ^a^	3.46 ± 0.11 ^ab^	6.93 ± 1.22 ^c^	4.16 ± 0.66 ^b^	4.83 ± 0.37 ^b^	6.94 ± 0.09 ^c^

* Starch was calculated as difference; TDF, total dietary fiber; IDF, insoluble dietary fiber; SDF, soluble dietary fiber; TEAC, Trolox equivalent antioxidant capacity. In the same row, data having different letters are significantly different (*p* < 0.05).

**Table 2:** Technological characterization of raw gnocchi.

	GF	GFR	GFB	C	CR	CB
L*	77.5 ± 2.3 ^c^	58.4 ± 1.4 ^b^	54.3 ± 3.2 ^a^	76.2 ± 1.7 ^c^	58.5 ± 2.9 ^b^	54.2 ± 2.3 ^a^
a*	−5.5 ± 0.5 ^b^	3.5 ± 0.2 ^e^	0.9 ± 0.2 ^d^	−0.6 ± 0.4 ^a^	3.3 ± 0.9 ^e^	0.5 ± 0.3 ^c^
b*	22.7 ± 2.3 ^d^	7.1 ± 0.6 ^b^	5.8 ± 1.0 ^a^	25.3 ± 1.6 ^e^	10.0 ± 0.8 ^c^	6.7 ± 0.8 ^ab^
Area (mm^2^)	447.0 ± 46.3 ^a^	539.4 ± 62.6 ^c^	510.6 ± 66.6 ^bc^	442.4 ± 36.5 ^a^	491.7 ± 63.4 ^b^	534.2 ± 69.1 ^c^
Width (mm)	18.7 ± 1.1 ^a^	22.0 ± 1.9 ^cd^	22.7 ± 2.6 ^d^	19.0 ± 1.0 ^a^	20.1 ± 1.8 ^b^	21.6 ± 1.6 ^c^
Length (mm)	30.8 ± 2.5 ^ab^	32.1 ± 2.7 ^bc^	30.1 ± 2.8 ^a^	29.9 ± 2.1 ^a^	31.8 ± 2.7 ^bc^	32.7 ± 3.3 ^c^

In the same row, data having different letters are significantly different (*p* < 0.05).

**Table 3:** Technological characterization of cooked gnocchi.

	GF	GFR	GFB	C	CR	CB
L*	69.6 ± 0.9 ^e^	52.2 ± 0.8 ^c^	44.7 ± 1.5 ^a^	64.3 ± 1.0 ^d^	51.7 ± 1.3 ^c^	47.1 ± 1.6 ^b^
a*	−6.7 ± 0.5 ^a^	4.4 ± 0.5 ^d^	1.5 ± 0.3 ^c^	−6.3 ± 0.3 ^b^	4.8 ± 0.4 ^e^	1.3 ± 0.3 ^c^
b*	21.7 ± 1.8 ^f^	8.1 ± 0.7 ^c^	5.1 ± 1.1 ^a^	20.2 ± 1.3 ^e^	10.0 ± 0.5 ^d^	6.9 ± 0.6 ^b^
Weight increase (g/100 g)	12.1 ± 1 ^b^	14.2 ± 1 ^c^	11.4 ± 1 ^a^	11.5 ± 1 ^ab^	14.1 ± 1 ^c^	16.4 ± 1 ^d^
Solid loss (g/100 g)	4.06 ± 0.51 ^a^	5.38 ± 1.23 ^b^	3.52 ± 0.70 ^a^	3.70 ± 0.60 ^a^	3.93 ± 0.61 ^a^	3.48 ± 0.73 ^a^
Hardness (N)	408 ± 13 ^f^	108 ± 2 ^a^	243 ± 8 ^c^	336 ± 17 ^e^	200 ± 18 ^b^	262 ± 8 ^d^

In the same row, data having different letters are significantly different (*p* < 0.05).

**Table 4:** Frequency mention (%) of Check-All-That-Apply (CATA) items for each cooked gnocchi sample.

CATA Items	Q	GF	GFR	GFB	C	CR	CB
Pleasant appearance	84.1	79.2	38.5	50.0	84.4	62.5	50.0
Unpleasant appearance	37.0	5.2	26.0	18.8	3.1	10.4	17.7
Pleasant taste	43.4	65.6	47.9	44.8	80.2	65.6	55.2
Unpleasant taste	55.1	1.0	18.8	26.0	1.0	5.2	13.5
Firm	25.2	42.7	20.8	29.2	44.8	37.5	25.0
Coarse	255.0	26.0	57.3	84.4	0.0	14.6	80.2
Rubbery to chew	45.3	53.1	19.8	28.1	49.0	49.0	25.0
Soft to chew	31.8	31.3	56.3	24.0	44.8	44.8	34.4
Grainy/pieces	182.0	9.4	44.8	63.5	1.0	18.8	71.9
Adhesive/Sticks to theet	139.9	14.6	71.9	29.2	12.5	58.3	18.8
Pleasant texture	63.9	56.3	20.8	26.0	63.5	33.3	31.3
Unpleasant texture	73.8	5.2	46.9	39.6	9.4	24.0	34.4

All values are significant at *p* < 0.0001.
